# Patterns of Adult Neuromyelitis Optica Spectrum Disorder Patients Compared to Multiple Sclerosis: A Systematic Review and Meta-Analysis

**DOI:** 10.7759/cureus.47565

**Published:** 2023-10-24

**Authors:** Mohammed Alqwaifly, Ahmed H Althobaiti, Nouf S AlAibani, Reemas Z Banjar, Rasil Sulaiman Alayed, Sara M Alsubaie, Aseel T Alrashed

**Affiliations:** 1 Department of Medicine, Unaizah College of Medicine and Medical Sciences, Qassim University, Buraydah, SAU; 2 Department of Neurology, King Saud Medical City, Riyadh, SAU; 3 College of Medicine, Qassim University, Buraydah, SAU; 4 College of Medicine, Umm Al-Qura University, Makkah, SAU; 5 College of Medicine, Almaarefa University, Riyadh, SAU

**Keywords:** patient characteristics, expanded disability status scale, meta-analysis, multiple sclerosis, neuromyelitis optica spectrum disorders

## Abstract

Neuromyelitis optica spectrum disorders (NMOSDs) are central nervous system inflammatory conditions, now recognized to involve the brain, often identified by aquaporin-4 (AQP4) antibodies. We aimed to summarize the characteristics of adult NMOSD patients compared to multiple sclerosis (MS). A computerized search was conducted on MEDLINE via PubMed, Web of Science, and ProQuest using the relevant keywords. Three independent reviewers performed two-stage screening and data extraction. The Review Manager 5.4 program (Cochrane Collaboration, Windows, London, UK) was used for the analysis. The Joanna Briggs Institute (JIB) tool was used for the quality of included studies. Twenty-three articles were included. NMOSD patients were associated with older age at presentation and higher Expanded Disability Status Scale (MD = 3.88, 95% CI: 1.80 to 5.97, P = 0.0003) and (MD = 1.15, 95% CI: 0.58 to 1.72, P < 0.0001), respectively. The risk of NMOSD in females was significantly higher than MS (OR = 2.21, 95% CI: 1.41 to 3.46, P = 0.0005). Patients with NMOSD were associated with a lower risk of extrapyramidal symptoms (OR = 0.26, 95% CI: 0.11 to 0.60, P < 0.01), brainstem involvement symptoms (OR = 0.32, 95% CI: 0.16 to 0.64, P < 0.01), and developing brain lesions compared to MS (OR = 0.08, 95% CI: 0.03 to 0.18, P < 0.00001). The current evidence suggests that both NMOSD and MS have different demographic, clinical, and lesion characteristics. There is a need for additional validation of the identified differences compared with MS due to the lack of long-term systematic imaging investigations in NMOSD.

## Introduction and background

Neuromyelitis optica spectrum disorders (NMOSDs) represent idiopathic inflammatory disorders of the central nervous system (CNS) that are manifested by severe attacks of optic neuritis (ON) and myelitis [1]. Brain involvement was not considered a diagnostic criterion of NMOSD until recently. In fact, the absence of brain involvement in magnetic resonance imaging (MRI) was previously regarded as a significant distinction in the NMOSD diagnosis [2]. However, brain abnormalities have been recognized in most NMOSD cases over the last two decades [3-5].

The discovery of aquaporin-4 antibodies (AQP4-abs) in 2004 has enhanced our understanding of NMOSD as it was a critical distinction from multiple sclerosis (MS) [6]. However, a small percentage of patients with NMOSD are seronegative for anti-AQP4-IgG, indicating a wide range of NMO spectrum disorders or related diseases [7]. Furthermore, the sensitivity and specificity of anti-AQP4-IgG assays differ. Although most modern assays work well in high-probability patients, when used for screening, the seropositive rate can change for assays with differing specificities [8].

Brain MRI of NMOSD patients has shown both symptomatic and asymptomatic brain involvement throughout the disease [4,6,7]. Most NMOSD brain abnormalities are located in areas with high AQP4 expression [2,5]. However, brain involvement can also be evident in brain sites where the expression of AQP-4 is relatively low [4]. NMOSD is associated with disease-specific lesions with the same appearance and location. Such lesions are identified using T2-weighted or fluid-attenuated inversion recovery (FLAIR) hyperintense, often revealing nonspecific white matter dots or patches [9,10].

NMOSD clinical features resemble other inflammatory or demyelinating CNS illnesses, especially in the early stages. Without appropriate preventive treatment that differs from standard MS treatment, they can cause significant impairment. Hence, determining the imaging characteristics of NMOSD must be a crucial step in diagnosing and treating the disease. This systematic review attempts to summarize the characteristics of adult NMOSD patients compared to MS. The primary objective of this systematic review and meta-analysis was to identify and analyze the distinct differences between NMOSD and MS, focusing on their brain and spinal cord lesion characteristics, as presented in MRI scans. Secondary objectives included understanding the difference between these two conditions in terms of demographics and clinical characteristics and assessing the quality of the included studies.

## Review

Methods

We adhered strictly to the "Preferred Reporting Items for Systematic Reviews and Meta-Analyses (PRISMA) extension statement for Network Meta-analyses of Health Care Interventions" [11]. Also, we used the "Cochrane Handbook for Systematic Reviews of Interventions" while implementing this study [12].

Eligibility Criteria

Studies must meet the following criteria to be included in this systematic review: observational (cohort, case-control, and cross-sectional) studies that include adult patients with NMOSD, studies that report the brain, spinal cord, or optic nerve MRI data, studies that compare NMOSD patients with MS patients, and studies that published in the English language. Reviews, editorials, abstracts, studies that include NMOSD pediatric participants, and studies that used non-conventional MRI were all excluded.

Information Source and Search Strategy

A literature search from inception to February 2022 was conducted using ProQuest, Web of Science, and MEDLINE via PubMed. The literature search was further updated in August 2023. The keywords used were ("Neuromyelitis Optica"[Mesh] OR NMO Spectrum Disorder OR Neuromyelitis Optica Spectrum Disorders OR Devic Neuromyelitis Optica) AND ("Multiple Sclerosis"(Mesh) OR Disseminated Sclerosis OR Sclerosis, Multiple OR Sclerosis, Disseminated) AND ("Magnetic Resonance Imaging"(Mesh) OR MRI OR MRI Brain OR Imaging, Magnetic Resonance). We identified the studies that contain these keywords in all fields. Search limits were used for document type, language, availability of full text, and peer review.

Search Strategy and Study Selection

EndNote was used to deduplicate the studies. Two independent reviewers (AHA and NSA) screened the titles and abstracts to check their relevance for full-text evaluation. Eligible studies were further evaluated by two independent reviewers (RZB and RSA) to assess the eligibility of full-text articles. Any disagreement between the reviewers was resolved by the discussion with the senior reviewer (MA).

Data Items and Data Collection Process

The primary outcome was the characteristics of brain MRI in NMOSD adult patients. These characteristics include demographic characteristics (age and gender), clinical characteristics (Expanded Disability Status Scale (EDSS), disease duration, and the number of relapses), clinical symptoms (headache, pyramidal, extrapyramidal, visual, brainstem, and seizures), and lesion characteristics (number, number per patient, location, morphology, diameter, and volume). The type of study, year of publication, number of participants, participants' age, gender, and MRI data were collected by two independent reviewers (SMA and ATA). Any disagreement between the reviewers was resolved by the discussion with the senior reviewer (MA).

Quality Assessment

Two independent reviewers appraised the quality of the included studies using the Joanna Briggs Institute (JBI) Critical Appraisal Checklist for observational studies. The JBI Critical Appraisal Checklist for cross-sectional studies consists of eight items about sample characteristics, measurement tools, and statistical analysis. The JBI Critical Appraisal Checklist for cohort studies includes 11 items in four domains: sample characteristics, measurement tools, statistical analysis, and follow-up [13].

Effect Measures and Synthesis Methods

The risk of developing brain and spinal cord lesions in both groups was calculated using the fixed-effect model of odds ratio (OR) with a 95% confidence interval (CI). For contentious outcomes such as age, disease duration, EDSS, and the number of relapses, we calculated the mean difference (MD) between both groups based on the inverse-variance model. Using the I^2^ statistic, we calculated the percentage of heterogeneity and inconsistency between studies, with values of 25%, 50%, and 75% deemed low, moderate, and high, respectively. The random-effect model was employed if the heterogeneity was significant and I^2^ > 50%; otherwise, the fixed-effect model was utilized. Review Manager 5.4.1. (Cochrane Collaboration, Windows, London, UK) was used for all statistical analyses. The sensitivity analysis by leave-one-out-of-the-analysis was performed. A subgroup analysis based on the study setting was conducted. Publication bias was assessed, and a funnel plot was generated for the forest plots that included 10 studies or more.

Results

Study Selection

**Figure 1:**
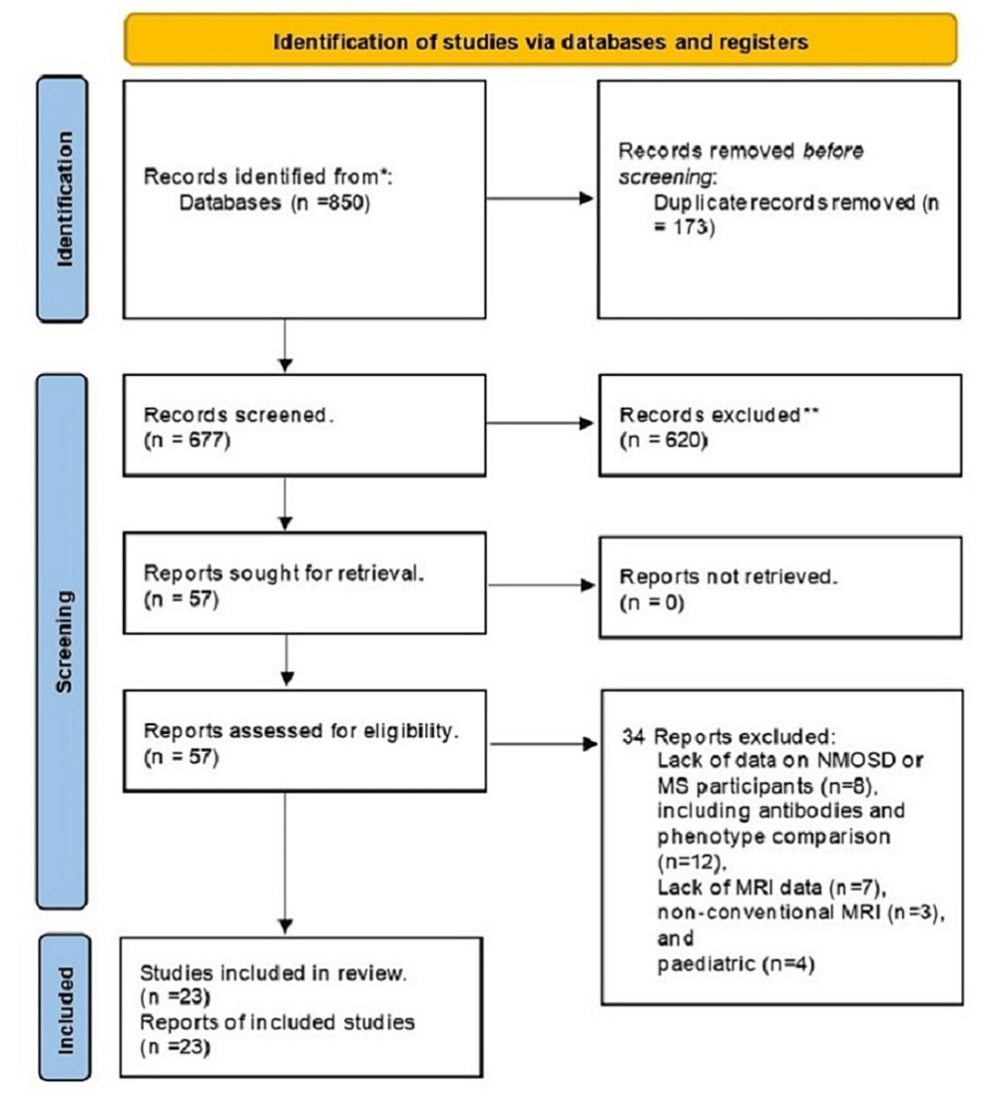
PRISMA flow diagram. PRISMA: Preferred Reporting Items for Systematic Reviews and Meta-Analyses.

The full-text manuscript of 57 studies was reviewed to assess eligibility. The initial search identified 850 manuscripts. After duplication, 173 manuscripts were eliminated. After assessing titles and abstracts, 620 articles were excluded due to a lack of relevance to the study question. Thirty-four manuscripts were excluded for several reasons, including lack of data on NMOSD or MS participants (n = 8), antibodies and phenotype comparison (n = 12), lack of MRI data (n = 7), and use non-conventional MRI (n = 3), and pediatric participants (n = 4). Finally, 23 manuscripts were eligible and included in this systematic review and meta-analysis [14-36] (Figure 1).

Characteristics of Included Studies

Nine studies were conducted in China, five in Korea, two in Japan, and one in India, France, Latin America, Malaysia, Taiwan, the UK, and the USA. Almost all of the included studies are cross-sectional except for two cohort studies. Table 1 summarizes the characteristics of the included studies.

**Table 1:** Summary of included studies. EDSS: Expanded Disability Status Scale; NR: not reported; NMOSD: neuromyelitis optica spectrum disorder; MS: multiple sclerosis; MRI: magnetic resonance imaging; FLAIR: fluid-attenuated inversion recovery; TSE: turbo spin echo; WI: weighted images; DTI: diffusion tensor imaging; MPRAGE: magnetization-prepared rapid acquisition gradient echo; ADC: apparent diffusion coefficient; STIR: short tau inversion recovery; FSE: fast spin echo; QSM: quantitative susceptibility mapping.

Study ID	Country	Design	Groups	Age, years	Female (%)	EDSS	Disease duration	Follow-up, months	Used methods
Lu et al., 2011 [34]	China	Cross-sectional	NMOSD (n = 23)	38.4 ± 12.5	18 (78.2%)	3.5 (1.5–6.5)	36.0 (15.0–84.0)	27.0 (15.0–41.0)	Brain MRI using a 1.5 T. Magnetic resonance spectroscopy. FLAIR, (TSE) T2 SE T1-weighted imaging.
			MS (n = 25)	37.0 ± 12.4	15 (60%)	3.0 (1.0–5.5)	24.0 (12.0–156.0)	24.0 (12.0–45.0)	
Viswanathan et al., 2013 [27]	Malaysia	Cross-sectional	NMOSD (n = 77)	NR	NR	NR	NR	NR	MRI using a 1.5 Tesla machine. Axial and sagittal views of T1- and T2-weighted images (WI), FLAIR images, and T1 pre- and post-gadolinium WI
			MS (n = 104)	28.6 ± 9.9	87 (83%)	2.71 ± 1.84	6.41 ± 5.23	NR	
Liao et al., 2014 [20]	Taiwan	Cross-sectional	NMOSD (n = 25)	37.8 ± 13.6	22 (88%)	NR	NR	NR	MRI: T1, T1-enhanced, T2-weighted, and FLAIR
			MS (n = 29)	33.7 ± 9.2	22 (75.86%)	NR	NR	NR	
Long et al., 2014 [14]	China	Cross-sectional	NMOSD (n = 47)	34.3 ± 14.0	45 (95.74%)	4.5 (1–10)	NR	NR	MRI using 1.5T MRI scanner: T1 with gadolinium enhancement for brain MRI
			MS (n = 37)	31.5 ± 12.2	20 (54.05%)	2.5 (0–6.5)	NR	NR	
Zhang et al., 2014 [29]	China	Cross-sectional	NMOSD (n = 23)	34.2 ± 10.2	22 (96.75%)	3.2 (1–8.5)	28.3 (11–91)	25.7 (14–50)	MRI using 1.5T MRI scanner. T1 with and without gadolinium enhancement and FLAIR
			MS (n = 60)	36.3 ± 12.1	33 (55%)	2.9 (1–10)	33.2 (9–192)	28.2 (18–56)	
Matthews et al., 2015 [33]	UK	Cohort	NMOSD (n = 18)	46 (20–76)	15 (83.33%)	4 (2–6)	57.5 (12–186)	NR	MRI using 3T. Structural three-dimensional T1 weighted scans for volumetric analysis with axial two-dimensional T2, proton density, and FLAIR imaging for lesions detection, 60-direction DTI, and myelin water imaging using the mcDESPOT multi-component technique
			MS (n = 15)	38 (22–62)	11 (73.33%)	2 (0–5)	72 (24–240) `	NR	
Buch et al., 2017 [21]	France	Cross-sectional	NMOSD with acute optic neuritis (n = 13)	30 (17–61)	12 (92%)	NR	NR	30.5 (2–71)	MRI was performed for all patients on 1.5T or 3T magnet and included at least FLAIR, T2-weighted (T2W), postgadolinium T1 (T1W Gd) imaging of the entire brain, and high resolution, 2–3 mm slices, coronal T2W and T1W.
			MS with acute optic neuritis (n = 20)	33 (18–62)	24 (75%)	NR	NR	34 (1–58)	
Fan et al., 2017 [15]	China	Cross-sectional	NMOSD (n = 55)	48.5 ± 11.6	42 (76.36%)	3.3 ± 2.0	5.91 ± 5.04	NR	MRI using a 3.0 T MR system. Axial T2-weighted turbo spin echo, 3D T1-weighted images sequence, 2D echo-planar DTI
			MS (n = 25)	43.3 ± 9.6	16 (64%)	2.7 ± 1.6	4.13 ± 5.81	NR	
Hyun et al., 2017 [32]	Korea	Cross-sectional	NMOSD (n = 91)	36 ± 7	82 (90%)	8 ± 5	2.7 ± 1.8	NR	MRI using a 3.0-T scanner: T1 and FLAIR image: 3D high-resolution sagittal FLAIR T2-weighted sequence
			MS (n = 52)	34 ± 7	30 (58%)	7 ± 4	1.9 ± 1.7	NR	
Lee et al., 2018 [16]	China	Cross-sectional	NMOSD (n = 13)	44.2 ± 13.4	11 (84.6%)	4 (2.5–6.5)	3.5 (2–4)	NR	MRI using a 3T scanner: brain T1-weighted (T1w) images acquired using 3D, T2-weighted (T2w) spin-echo images
			MS (n = 17)	41.0 ± 9.4	12 (70.6%)	6 (2.5–11)	2 (1.5–4)	NR	
Liu et al., 2018 [18]	China	Cohort	NMOSD = 25	35.6 ± 9.9	21 (84%)	4.0 ± 2.8	3.5 (0.5–7)	NR	MRI using a 1.5T: T2-weighted turbo spin-echo, FLAIR, sagittal 3D T1-weighted MPRAGE.
			MS = 20	35.5 ± 10.6	15 (75%)	3.7 ± 2.5	2.5 (0–6.5)	NR	
Tatekawa et al., 2018 [26]	Japan	Cross-sectional	NMOSD (n = 89)	77 ± 86.5	68 (76.4%)	4 (0–11)	6 (2–7.5)	NR	MRI using 1.5T or 3T scanners: T2-weighted FSE images, along with FLAIR and/or T1-weighted images with/without gadolinium enhancement
			MS (n = 89)	68 ± 76.4	77 (86.5%)	2 (1–7)	2 (1–3)	NR	
Wei et al., 2018 [28]	China	Cross-sectional	NMOSD (n = 49)	44.7 ± 17.0	38 (77.55%)	6.5 (2.0–8.5)	18.9 ± 8.8	NR	NR
			MS (n = 12)	37.4 ± 16.0	9 (75.0%)	3.5 (2.0–5.0)	22.4 ± 7.3	NR	
Hyun et al., 2019 [30]	Korea	Cross-sectional	NMOSD (n = 64)	NR	45 (70.31%)	NR	NR	NR	MRI using a 1.5- or 3.0-T: The brain lesion distribution criteria were defined as at least one lesion with the following characteristic(s): adjacent to the body of the lateral ventricle and in the inferior temporal lobe; juxtacortical S-shaped U-fiber; or Dawson’s finger type lesions.
			MS (n =53)	NR	45 (84.90%)	NR	NR	NR	
Lee et al., 2019 [19]	Korea	Cross-sectional	NMOSD (n = 26)	43 ± 15	23 (88.46%)	2 (1.5–4)	NR	NR	MRI using 1.5T or 3.0T scanners: DWI, FLAIR, T1-, and T2-weighted gradient-echo axial imaging.
			MS (n = 42)	37 ± 12	25 (59.52%)	2 (1.5–3)	NR	NR	
Banks et al., 2020 [17]	USA	Cross-sectional	NMOSD (n = 30)	45 (6–72)	27 (90%)	NR	NR	NR	Axial T2-weighted, fluid-attenuated inversion recovery (FLAIR) and axial T1-postgadolinium sequences were predominantly used for analysis.
			MS (n = 30)	36 (16–65)	22 (73%)	NR	NR	NR	
Contentti et al., 2020 [31]	Latin America	Cross-sectional	NMOSD = 94	37.6 ± 14.6	76 (80.8%)	NR	NR	NR	MRI using 3 Tesla: Brain (coronal, axial, and sagittal) and cervical and thoracic spinal cord (axial and sagittal)
			MS = 188	34.09 ± 11.7	142 (75.5%)	NR	NR	NR	
Jang et al., 2020 [24]	Korea	Cross-sectional	NMOSD (n = 21)	49.0 ± 14.4	19 (90.47%)	3.00 (1.0–3.5)	20.9 (0.8–109.4)	NR	MRI using 3-T MRI: QSM, conventional T1-weighted images, two-weighted images with and without FLAIR
			MS (n = 32)	35.0 ± 10.6	25 (78.13%)	1.75 (1.0–2.5)	33.1 (3.2–66.6)	NR	
Kim et al., 2020 [36]	China	Cross-sectional	NMOSD (n = 125)	41.7 ± 13.7	112 (89.6%)	3.3 ± 1.8	5.3 ± 5.6	NR	MRI using 1.5T or 3.0T scanners: 2D FLAIR sequences
			MS (n = 213)	33.1 ± 12.3	158 (74.18%)	2.4 ± 1.8	7.6 ± 6.6	NR	
Duan et al., 2021 [25]	China	Cross-sectional	NMOSD (n = 38)	37.7 ± 11.9	32 (84%)	3.5 (2–4.5)	1.4 (0.8–3.2)	NR	Brain MRI using 3.0 Tesla: FLAIR, 3D T1 and diffusion tensor imaging (DTI)
			MS (n = 37)	33.8 ± 11.2	23 (62%)	2.5 (1–3)	3 (1–5.3)	NR	
Kumar et al., 2021 [23]	India	Cross-sectional	NMOSD (n = 20)	28.3 ± 11.21	14 (70%)	2.65 ± 2.43	2.12 ± 2.22	NR	Brain MRI using 3 Tesla: T1, T2, FLAIR, diffusion-weighted imaging (DWI), apparent diffusion coefficient (ADC) mapping, STIR, gradient echo (GRE).
			MS (n = 40)	34 ± 7.34	24 (60%)	3.23 ± 1.94	7 ± .14	NR	
Kim et al., 2022 [22]	Korea	Cross-sectional	NMOSD (n = 20)	46.16 ± 12.33	17 (85%)	3.31 ± 2.78	46.2 ± 45.1	NR	Brain MRI using 3.0 T: T2 FLAIR imaging, T1-weighted imaging, and DTI were acquired
			MS (n = 24)	34.45 ± 7.94	12 (50%)	1.77 ± 1.43	59.6 ± 71.5	NR	
Masuda et al., 2022 [35]	Japan	Cross-sectional	NMOSD (n = 51)	52.0 ± 18.0	46 (90.2%)	3.0 ± 4.0	8.0 ± 13.0	NR	Brain MRI using a 1.5-Tesla: FLAIR and 3D T1 weighted images
			MS (n = 85)	42.0 ± 13.0	66 (77.6%)	2.5 ± 3.0	11.0 ± 10.0	NR	

Quality Assessment

Based on the JBI Critical Appraisal Checklist for observational studies, six out of 21 cross-sectional studies showed high quality, 14 showed moderate quality, and one showed low quality. Regarding cohort studies, both studies showed a high quality. See Appendix (Tables 5, 6), which presents the detailed JBI checklist and our critical appraisal results.

Meta-analysis

Demographic and Clinical Characteristics

**Figure 2:**
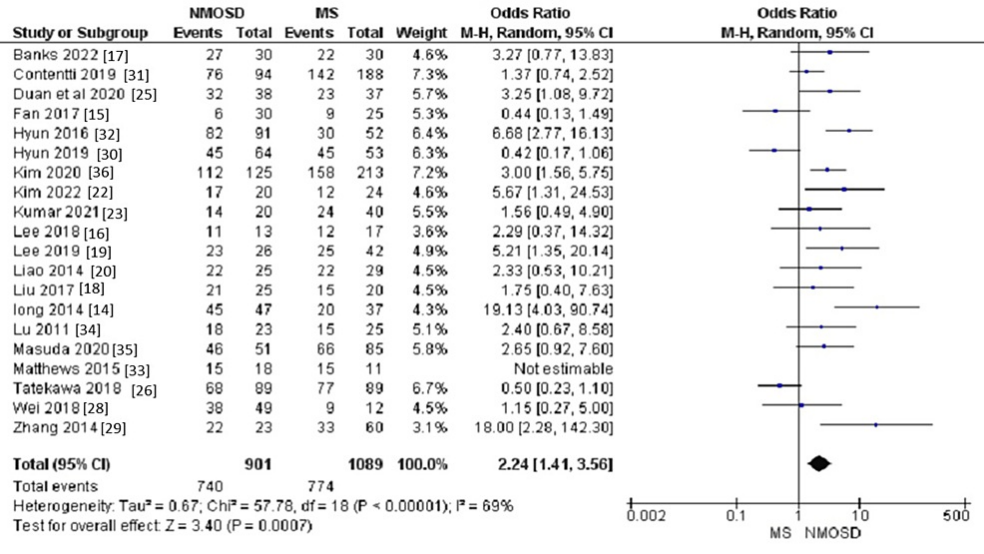
Forest plot of the difference between NMOSD and MS in terms of female gender. NMOSD: neuromyelitis optica spectrum disorder; MS: multiple sclerosis.

Gender: The pooled analysis of 20 studies showed that NMOSD patients are more likely to be females compared to MS patients (OR = 2.21, 95% CI: 1.41 to 3.46, P = 0.0005, Figure 2). Heterogeneity within this analysis was moderate (I² = 67%, p<0.001). After the application of sensitivity analysis, heterogeneity can be resolved (I^2 ^= 34%, p = 0.10) by excluding four studies: Contentti et al. [31], Fan et al. [15], Hyun et al. [30], and Tatekawa et al. [26], with an effect size of (OR = 3.32, 95% CI: 2.39 to 4.61, P<0.00001). 

**Figure 3:**
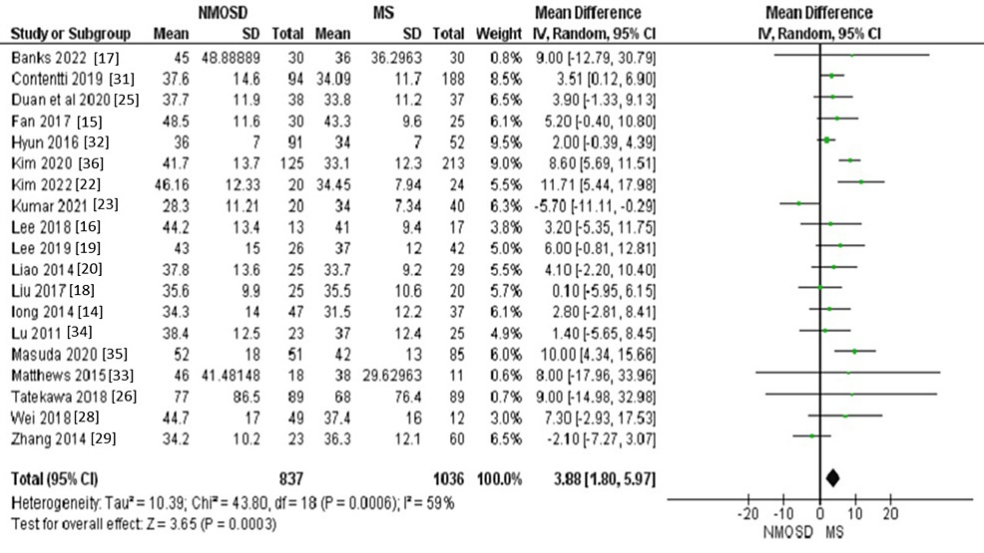
Forest plot of the difference between NMOSD and MS in terms of age. NMOSD: neuromyelitis optica spectrum disorder; MS: multiple sclerosis.

Age: Regarding the patients' age, patients with NMOSD seem to be older than patients with MS (MD = 3.88, 95% CI: 1.80 to 5.97, P = 0.0003, Figure 3). This outcome had a moderate level of heterogeneity (I² = 59%, p < 0.001), which could be resolved after the exclusion of Kim et al., 2020 [36], Kumar et al., 2021 [23], and Zhang et al., 2014 [29] (MD = 4.42, 95% CI: 3.16 to 5.67, P < 0.00001; I^2 ^= 25%, p = 0.17).

**Figure 4:**
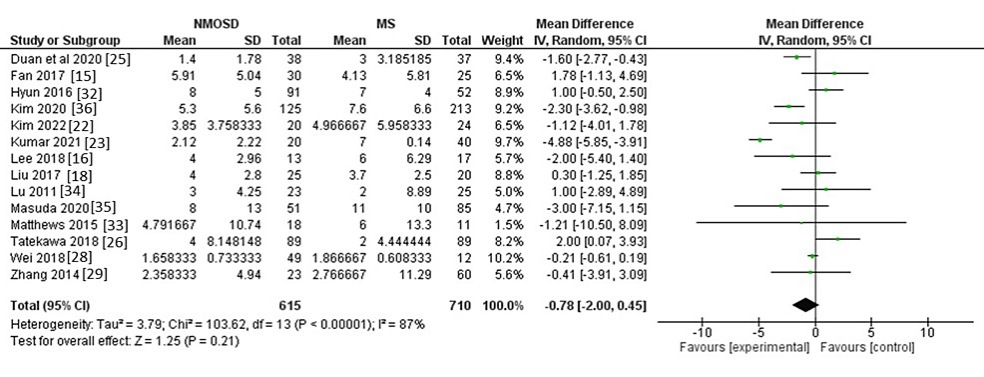
Forest plot of the difference between NMOSD and MS in terms of disease duration. NMOSD: neuromyelitis optica spectrum disorder; MS: multiple sclerosis.

Disease duration: We identified 14 studies that reported relevant data for disease duration, involving a total of 1,325 participants. We did not find evidence of a clear difference between the two groups regarding disease duration (MD = -0.78, 95% CI: -2 to 0.45, P = 0.21, Figure 4). The pooled data were heterogeneous (I^2 ^= 87%, p < 0.001).

**Figure 5:**
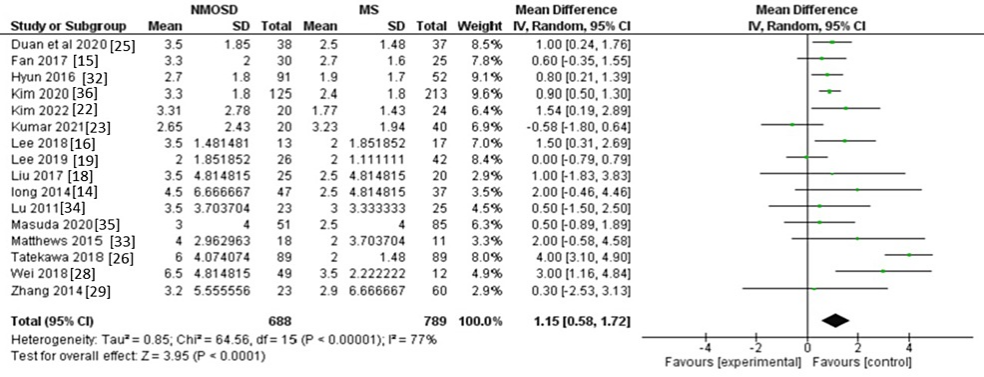
Forest plot of the difference between NMOSD and MS in terms of EDSS. NMOSD: neuromyelitis optica spectrum disorder; MS: multiple sclerosis; EDSS: Expanded Disability Status Scale.

EDSS: Sixteen studies provided adequate data for EDSS, with a total of 1,477 participants. Patients with NMOSD were associated with higher EDSS compared to MS patients (MD = 1.15, 95% CI: 0.58 to 1.72, P < 0.0001, Figure 5).

**Figure 6:**
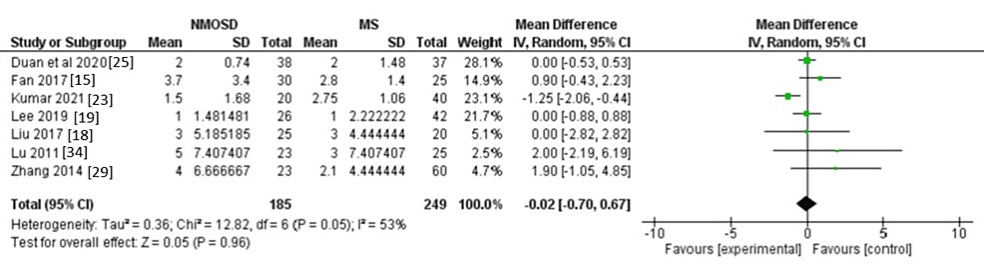
Forest plot of the difference between NMOSD and MS in terms of the number of relapses. NMOSD: neuromyelitis optica spectrum disorder; MS: multiple sclerosis.

Number of relapses: We identified seven studies that reported relevant data for the number of relapses, involving 434 participants. However, there was not a clear difference between NMOSD and MS (MD = -0.02, 95% CI: -0.7 to 0.67, P = 0.96, Figure 6). The heterogeneity of effects for this outcome was moderately high (I² = 53%, p = 0.05).

Clinical Symptoms

The pooled analysis showed that patients with NMOSD were associated with a higher risk of optic neuritis (OR = 4.89, 95% CI: 2.67 to 8.95, P < 0.00001), myelitis (OR = 9.55, 95% CI: 4.60 to 19.84, P < 0.00001), headache (OR = 2.39, 95% CI: 1.12 to 5.07, P < 0.05), sensory affection symptoms (OR = 6.68, 95% CI: 2.35 to 19.02, P < 0.001), and visual involvement symptoms (OR = 63.24, 95% CI: 7.42 to 538.88, P < 0.001). On the other hand, NMOSD patients were associated with a lower risk of extrapyramidal symptoms (OR = 0.26, 95% CI: 0.11 to 0.60, P < 0.01) and brainstem involvement symptoms (OR = 0.32, 95% CI: 0.16 to 0.64, P < 0.01). Both groups demonstrated a comparable risk in terms of pyramidal symptoms (OR = 1.5, 95% CI: 0.79 to 2.85, P = 0.22), encephalopathy (OR = 0.84, 95% CI: 0.20 to 3.48, P = 0.81), and seizure (OR= 0.66, 95% CI: 0.07 to 6.48, P = 0.72), see Table 2.

**Table 2:** Clinical symptoms. OR: Odds ratio; M-H: Mantel Haenszel Test; MD: mean difference; CI: confidence interval; NMOSD: neuromyelitis optica spectrum disorder; MS: multiple sclerosis.

Outcome	Studies	Participants	NMOSD vs. MS	Statistical method	Effect estimate	Heterogeneity
Optic neuritis	2	211	67 (75.28%) vs. 46 (37.70%)	OR (M-H, Fixed)	4.89 (2.67, 8.95)	I^2 ^= 50%, p = 0.16
Myelitis	3	206	70 (83.33%) vs. 45 (36.89%)	OR (M-H, Fixed)	9.55 (4.60, 19.84)	I^2 ^= 0%, p = 0.41
Encephalopathy	3	206	3 (3.57%) vs. 5 (4.10%)	OR (M-H, Fixed)	0.84 (0.20, 3.48)	I^2 ^= 0%, p = 0.92
Headache	3	199	18 (25.0%) vs. 18 (14.17%)	OR (M-H, Fixed)	2.39 (1.12, 5.07)	I^2 ^= 37%, p = 0.20
Seizure	3	151	0 (0.00%) vs. 2 (1.96%)	OR (M-H, Fixed)	0.66 (0.07, 6.48)	I^2 ^= 0%, p = 0.84
Pyramidal	4	253	72 (74.23%) vs. 108 (69.23%)	OR (M-H, Fixed)	1.50 (0.79, 2.85)	I^2 ^= 50%, p = 0.11
Sensory	3	185	67 (94.37%) vs. 76 (66.67%)	OR (M-H, Fixed)	6.68 (2.35, 19.02)	I^2 ^= 0%, p = 0.62
Extrapyramidal	3	189	11 (14.86%) vs. 41 (35.65%)	OR (M-H, Fixed)	0.26 (0.11, 0.60)	I^2 ^= 26%, p = 0.26
Visual	2	131	46 (100%) vs. 40 (47.06%)	OR (M-H, Fixed)	63.24 (7.42, 538.88)	I^2 ^= 0%, p = 0.42
Brainstem	3	191	44 (57.89%) vs. 90 (78.26%)	OR (M-H, Fixed)	0.32 (0.16, 0.64)	I^2 ^= 0%, p = 0.73

Brain Lesion Characteristics

The random-effect model showed that patients with NMOSD were associated with a lower risk of developing brain lesions compared to MS (OR = 0.08, 95% CI: 0.03 to 0.18, P < 0.00001), including cortical/juxtacortical lesions (OR = 0.45, 95% CI: 0.21 to 0.94, P < 0.05), subcortical white matter lesions (OR = 0.47, 95% CI: 0.26 to 0.88, P < 0.05), periventricular white matter lesions (OR = 0.01, 95% CI: 0 to 0.05, P < 0.00001), corpus callosum lesions (OR = 0.06, 95% CI: 0.03 to 0.14, P < 0.00001), thalamus lesions (OR = 0.43, 95% CI: 0.19 to 0.99, P < 0. 05), brainstem lesions (OR = 0.25, 95% CI: 0.16 to 0.38, P < 0.00001), midbrain lesions (OR = 0.46, 95% CI: 0.23 to 0.93, P < 0.05), cerebellar peduncles lesions (OR = 0.18, 95% CI: 0.08 to 0.40, P < 0.0001), cerebellum lesions (OR = 0.07, 95% CI: 0.02 to 0.29, P < 0.001), cerebrum lesions (OR = 0.28, 95% CI: 0.11 to 0.69, P < 0.01), and pons lesions (OR = 0.27, 95% CI: 0.12 to 0.60, P < 0.01). On the other hand, NMOSD patients were associated with a significantly higher risk of spinal cord lesions (OR = 5.84, 95% CI: 1.61 to 21.25, P < 0.01), optic nerve lesions (OR = 2.27, 95% CI: 1.35 to 3.82, P < 0.01), deep gray matter lesions (OR = 3.82, 95% CI: 2.28 to 6.38, P < 0.00001), deep white matter lesions (OR = 2.53, 95% CI: 2.15 to 2.97, P < 0.00001), hypothalamus lesions (OR = 8.08, 95% CI: 3.04 to 21.46, P < 0.0001), and medulla oblongata (OR = 3.51, 95% CI: 1.9 to 6.48, P < 0.0001). Both groups had a comparable risk of basal ganglia lesions (OR = 0.64, 95% CI: 0.30 to 1.38, P = 0.25) and lesions adjacent to the body of the lateral ventricle (OR = 1.03, 95% CI: 0.51 to 2.08, P = 0.95). In terms of gadolinium enhancement, we found a significant difference between the NMOSD and MS groups (OR = 0.37, 95% CI: 0.21 to 0.67, P < 0.001). Regarding lesion morphology, patients with NMOSD were associated with a lower risk of Dawson’s finger type lesions (OR = 0.03, 95% CI: 0.01 to 0.06, P < 0.00001) and S or U shape lesions (OR = 0.19, 95% CI: 0.09 to 0.39, P < 0.00001). We did not find evidence of a clear difference between the two groups in terms of lesion diameter (MD = 1.36, 95% CI: -1.76 to 4.47, P = 0.39), thalamic volume (MD = 0.7, 95% CI: -0.08 to 1.49, P = 0.08), or number of lesions per patient (MD = -0.64, 95% CI: -1.60 to 0.32, P = 0.19), see Table 3.

**Table 3:** Characteristics of brain lesions. OR: Odds ratio; M-H: Mantel Haenszel Test; MD: mean difference; CI: confidence interval; NMOSD: neuromyelitis optica spectrum disorder; MS: multiple sclerosis.

Outcomes	Studies	Participants	NMOSD vs. MS	Statistical methods	Effect size	Heterogeneity
Number of lesions	5	482	166 (76.85%) vs. 261 (98.12%)	OR (M-H, Fixed, 95% CI)	0.08 (0.03, 0.18)	I^2^: 0%, p = 0.54
Cortical/juxtacortical lesions	3	182	21 (30.88%) vs. 44 (46.81%)	OR (M-H, Fixed, 95% CI)	0.45 (0.21, 0.94)	I^2^: 27%, p = 0.25
Subcortical white matter lesions	4	3013	122 (11.15%) vs. 305 (15.89%)	OR (M-H, Random, 95% CI)	0.47 (0.26, 0.88)	I^2^: 68%, p = 0.03
Deep gray matter lesions	2	2641	48 (5.14%) vs. 23 (1.35%)	OR (M-H, Fixed, 95% CI)	3.82 (2.28, 6.38)	I^2^: 0%, p = 0.34
Deep white matter lesions	3	2718	648 (66.74%) vs. 756 (43.32%)	OR (M-H, Fixed, 95% CI)	2.53 (2.15, 2.97)	I^2^: 0%, p = 0.17
Periventricular white lesions	3	183	21 (25.93%) vs. 99 (97.06%)	OR (M-H, Fixed, 95% CI)	0.01 (0.00, 0.05)	I^2^: 0%, p = 0.54
Corpus callosum lesions	4	237	9 (8.49%) vs. 77 (58.78%)	OR (M-H, Fixed, 95% CI)	0.06 (0.03, 0.14)	I^2^: 0%, p = 0.53
Basal ganglia lesions	2	129	20 (31.75%) vs. 28 (42.42%)	OR (M-H, Fixed, 95% CI)	0.64 (0.30, 1.38)	I^2^: 64%, p = 0.10
Thalamus lesions	2	158	18 (29.51%) vs. 55 (56.70%)	OR (M-H, Fixed, 95% CI)	0.43 (0.19, 0.99)	I^2^: 0%, p = 0.76
Hypothalamus lesions	3	221	27 (28.42%) vs. 6 (4.76%)	OR (M-H, Fixed, 95% CI)	8.08 ((3.04, 21.46)	I^2^: 0%, p = 0.48
Brain stem lesions	4	595	39 (16.18%) vs. 138 (39.09%)	OR (M-H, Fixed, 95% CI)	0.25 (0.16, 0.38)	I^2^: 0%, p = 0.47
Midbrain lesions	3	183	21 (23.08%) vs. 34 (36.96%)	OR (M-H, Fixed, 95% CI)	0.46 (0.23, 0.93)	I^2^: 50%, p = 0.14
Medulla oblongata lesions	4	237	54 (46.55%) vs. 28 (23.14%)	OR (M-H, Fixed, 95% CI)	3.51 (1.90, 6.48)	I^2^: 30%, p = 0.12
Cerebellar peduncles lesions	3	183	13 (14.29%) vs. 39 (42.39%)	OR (M-H, Fixed, 95% CI)	0.18 (0.08, 0.40)	I^2^: 30%, p = 0.24
Cerebellum lesions	3	313	1 (0.68%) vs. 29 (17.47%)	OR (M-H, Fixed, 95% CI)	0.07 (0.02, 0.29)	I^2^: 0%, p = 0.10
Cerebrum lesions	2	109	26 (36.11%) vs. 21 (56.76%)	OR (M-H, Fixed, 95% CI)	0.28 ((0.11, 0.69)	I^2^: 12%, p = 0.28
Pons lesions	2	108	24 (45.28%) vs. 42 (76.36%)	OR (M-H, Fixed, 95% CI)	0.27 (0.12, 0.60)	I^2^: 50%, p = 0.10
Spinal cord lesions	5	560	176 (74.89%) vs. 141 (43.38%)	OR (M-H, Random, 95% CI)	5.84 (1.61, 21.25)	I^2^: 83%, p < 0.01
Optic nerve lesions	3	378	57 (35.40%) vs. 34 (15.81%)	OR (M-H, Fixed, 95% CI)	2.27 (1.35, 3.82)	I^2^: 53%, p = 0.12
Lesions adjacent to the body of the lateral ventricle	2	366	15 (10.64%) vs. 24 (10.67%)	OR (M-H, Fixed, 95% CI)	1.03 (0.51, 2.08)	I^2^: 34%, p = 0.22
Dawson’s finger type	3	357	10 (7.58%) vs. 167 (74.22%)	OR (M-H, Fixed, 95% CI)	0.03 (0.01, 0.06)	I^2^: 0%, p = 0.50
S or U shape lesions	5	706	38 (12.26%) vs. 184 (46.46%)	OR (M-H, Random, 95% CI)	0.19 (0.09, 0.39)	I^2^: 63%, p = 0.03
Number of lesions per patient	3	309	-	MD (IV, Fixed, 95% CI)	-0.64 (-1.60, 0.32)	I^2^: 1%, p = 0.36
Lesion diameter	4	377	-	MD (IV, Random, 95% CI)	1.36 (-1.76, 4.47)	I^2^: 77%, p = 0.005
Thalamic volume	2	188	-	MD (IV, Random, 95% CI)	0.70 (-0.08, 1.49)	I^2^: 77%, p = 0.04

Spinal Cord Lesion Characteristics

The pooled fixed-effect model showed that there was no significant difference between both groups in terms of the number of spinal cord lesions per patient (MD = -0.03, 95% CI: -0.37 to 0.31, P = 0.87). However, the length of the spinal lesions was significantly higher in the NMOSD groups than in the MS group (MD = 3.44 mm, 95% CI: 2.00 to 4.88, P < 0.00001). There was no significant difference between the two groups in terms of central lesions (OR = 3.61, 95% CI: 0.59 to 22.16, P = 0.17); however, the NMOSD group was associated with a lower risk of peripheral lesions (OR = 0.08, 95% CI: 0.01 to 0.91, P = 0.04). Concerning the region, there was no significant difference between the two groups in terms of cervical lesions (OR = 0.86, 95% CI: 0.49 to 1.50, P = 0.59) and thoracic lesions (OR = 0.64, 95% CI: 0.06 to 6.83, P = 0.71). On the other hand, the NMOSD group was associated with a higher risk of cervicothoracic lesions (OR = 2.62, 95% CI: 1.45 to 4.74, P = 0.001). Regarding the morphology, atrophic lesions were found to be significantly higher in the NMOSD group than in the MS group (OR = 7.93, 95% CI: 1.88 to 33.52, P = 0.005), see Table 4.

**Table 4:** Spinal cord lesions. OR: odds ratio; M-H: Mantel Haenszel Test; MD: mean difference; CI: confidence interval; NMOSD: neuromyelitis optica spectrum disorder; MS: multiple sclerosis.

Outcomes	Studies	Participants	NMOSD vs. MS	Statistical methods	Effect size	Heterogeneity
Number of lesions	2	223	-	MD (IV, Fixed, 95% CI)	-0.03 (-0.37, 0.31)	I^2^: 0; p = 0.34
Length of lesion	3	126	-	MD (IV, Fixed, 95% CI)	3.44 (2.00, 4.88)	I^2^: 0; p = 0.78
Central location	2	359	102 (61.45%) vs. 68 (35.23%)	OR (M-H, Random, 95% CI)	3.61 (0.59, 22.16)	I^2^: 92; p < 0.001
Peripheral location	2	359	14 (8.43%) vs. 100 (51.81%)	OR (M-H, Random, 95% CI)	0.08 (0.01, 0.91)	I^2^: 91; p < 0.001
Cervical region lesions	2	215	36 (36.00%) vs. 46 (40.00%)	OR (M-H, Random, 95% CI)	0.86 (0.49, 1.50)	I^2^: 0; p = 0.90
Cervicothoracic region lesions	2	215	46 (46.00%) vs. 27 (23.48%)	OR (M-H, Random, 95% CI)	2.62 (1.45, 4.74)	I^2^: 0; p = 0.70
Thoracic region lesions	2	215	10 (10.00%) vs. 10 (8.70%)	OR (M-H, Random, 95% CI)	0.64 (0.06, 6.83))	I^2^: 79; p = 0.03
Atrophy	2	359	43 (25.90%) vs. 8 (4.15%)	OR (M-H, Random, 95% CI)	7.93 (1.88, 33.52)	I^2^: 68; p = 0.08
Swelling	2	359	43 (25.90%) vs. 8 (4.15%)	OR (M-H, Random, 95% CI)	13.35 (0.24, 738.60)	I^2^: 87; p = 0.006

Subgroup Analysis

A subgroup analysis has been conducted according to the study country; the studies were divided into two groups: Chinese studies and other countries. The subgroup analysis showed that the patients with NMOSD were more likely to be females compared to MS in China (OR = 2.19, 95% CI: 1.05 to 4.57; p = 0.04) and other countries (OR = 2.28, 95% CI: 1.27 to 4.08; p = 0.006). Moreover, patients with NMOSD were more likely to be older than those with MS (China: MD = 3.50, 95% CI: 0.68 to 6.31; p = 0.01, other countries: MD = 4.42, 95% CI: 1.12 to 7.73; p = 0.0003). Regarding EDSS, it was significantly higher in Chinese patients with NMOSD than MS (MD = 0.88, 95% CI: 0.47 to 1.29; p < 0.0001); however, in the other countries, there was no significant difference between NMOSD and MS in terms if EDSS (MD = 1.37, 95% CI: -0.06 to 2.79; p = 0.06).

Publication Bias

**Figure 7:**
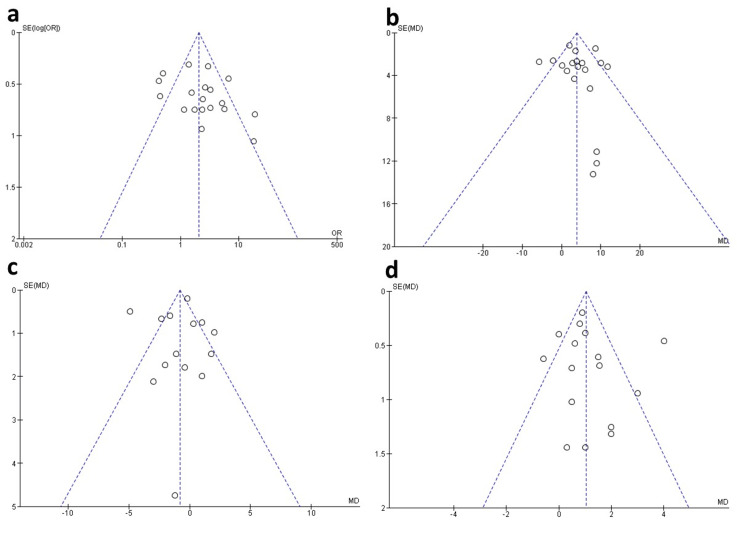
Funnel plot of publication bias. The figure shows the funnel plot of (a) gender, (b) age, (c) disease duration, and (d) EDSS. EDSS: Expanded Disability Status Scale.

In terms of gender comparison, Egger’s regression and Begg-Mazumdar Rank correlation showed no significant risk of bias in terms of gender (1.77; p = 0.077 and 0.189; p = 0.260; Figure 7a), age (-0.652; p = 0.514 and -0.029; p = 0.890; Figure 7b), disease duration (-0.135, p = 0.892, and -0.128; p = 0.590; Figure 7c), and EDSS (-0.296, p = 0.767, and 0.08; p = 0.690; Figure 7d), respectively. 

Discussion

In this systematic review and meta-analysis, our findings showed that patients with NMOSD were more likely to be females of older age compared to MS patients. Moreover, patients with NMOSD were associated with higher EDSS than MS patients. Regarding clinical symptoms, optic neuritis, myelitis, headache, sensory affection symptoms, and visual involvement are the most commonly reported symptoms in patients with NMOSD. In addition, patients with NMOSD were associated with a lower risk of developing brain lesions compared to MS patients; however, spinal cord lesions, optic nerve lesions, deep gray matter lesions, deep white matter lesions, hypothalamus lesions, and medulla oblongata lesions were more common in NMOSD patients than MS patients. Regarding lesion morphology, patients with NMOSD had a lower risk of Dawson's finger-type lesions and S or U-shaped lesions.

Reports indicate that between 43% and 70% of individuals with NMOSD present with preexisting brain MRI abnormalities [37,38]. Since the first brain MRI investigations in NMOSD, certain individuals have shown anomalies in the white matter that were neither clinically apparent nor diagnostic. The development of AQP4-IgG assays revealed that many people with NMOSD had MRI abnormalities in their brains, most often in regions where AQP4 expression was high [39]. In contrast, abnormalities presented themselves in regions of the brain where AQP4 expression was relatively low. The majority of lesions in NMOSD are small, indistinct spots and patches of hyperintensity in the subcortical and deep white matter on T2-weighted or fluid-attenuated inversion recovery sequences. However, there are certain lesions that have a location or appearance that is unique to NMOSD [37,38,40]. Inconsistencies in these results may be due to the fact that brain MRI abnormalities tend to occur more often as the disease progresses.

In clinical settings, MS is often considered a possible alternative diagnosis for NMOSD. The prognosis and treatment for each disease are different, and certain MS treatments may actually make NMOSD worse, so understanding the distinction between the two is crucial [41-43]. It is crucial to enhance the tools and analyses used to differentiate between these diseases for early and accurate diagnosis. Similarities and differences between the two diseases may shed light on the various pathogenic processes. Patients with NMOSD may be screened for the disease using a particular marker (serum anti-AQP4 antibodies), while the same cannot be said for MS. Different inclusion and exclusion criteria, such as whether or not patients with NMOSD had to test positive for the anti-AQP4 antibody, have been employed in studies comparing NMOSD and MS, which may have led to different findings. Different tests for anti-AQP4 antibodies have varying degrees of sensitivity, and this variability, together with variations in the length of follow-up, may contribute to contradictory findings. Patients who tested positive for anti-AQP4 antibody did not have significantly different lesion distributions from those with MS, according to lesion probability maps [44]. However, several diagnostic markers have been established on MS brain MRI that are both sensitive and specific, such as the presence of S-shaped U-fiber lesions, Dawson fingers lesions, inferior temporal lobe lesions, and lesions adjacent to the lateral ventricle. With the exception of a single Japanese investigation on NMO pathology [45], imaging sensitive to cortical lesions has shown that these lesions are not present in NMO, although they occur in most individuals with MS [46,47]. Lesions characteristics of MS are often seen around the central venule on high-intensity MRI in >80% of cases [48,49]. This is seen less often in NMO lesions, with reports ranging from 9% to 35% of cases, possibly pointing to distinct pathogenic processes [46,48,49]. There seems to be a difference in the prevalence of silent lesions between these disorders. Clinically silent MRI lesions are more common in MS patients and occur less often in those with NMOSD. There is a need for additional validation of the identified cross-sectional differences compared with MS due to the lack of long-term systematic imaging investigations in NMOSD. In order to further increase the sensitivity and specificity, it may be beneficial to develop algorithms that use the brain criteria established by Matthews et al. in conjunction with imaging aspects of the spinal cord and optic nerve and maybe non-conventional imaging [44].

ON of NMOSD is associated with gadolinium enhancement on T1-weighted sequences, optic nerve hyperintensities on T2-weighted sequences, and nonspecific thickening of the optic nerve sheath, according to MRI studies [50,51]. However, these results are not considered diagnostic of NMOSD since they were previously observed in the ON of MS [52]. Recent research has compared MS and NMOSD concerning the MRI characteristics of the optic nerve lesions in both [53,54]. NMOSD has been associated with increased posterior optic nerve involvement (including chiasm) and the simultaneous development of bilateral disease [53,54]. Thus, in the proper clinical setting, we should suspect the diagnosis of NMOSD when long-segment inflammation of the optic nerve is present, especially when bilateral and extending posteriorly into the optic chiasm.

Regarding spinal cord lesions, our findings showed that the length of the spinal lesions was significantly higher in the NMOSD groups than in the MS group. Moreover, the NMOSD group was associated with a lower risk of peripheral lesions and a higher risk of cervicothoracic lesions. Regarding morphology, atrophic lesions were significantly higher in the NMOSD group than in the MS group. Hyperintensity on T2-weighted sequences and hypo-intensity on T1-weighted sequences describe the inflammatory process of NMOSD on MRI of the spinal cord. Preferential involvement of the central grey matter has been observed in studies of MRIs of the spinal cord, and these abnormalities are more common in the cervical and upper thoracic spinal cord segments than in the lower thoracic and lumbar regions [55,56]. AQP4 is mainly found in the gray matter of the spinal cord and the glial cell processes next to the ependymal cells of the central canal. It is found in the white matter of the spinal cord to a lesser extent [57]. Longitudinally extensive transverse myelitis (LETM) is the most recognizable form of NMOSD, and it is characterized as a lesion that spans three or more adjacent vertebrae and affects the spinal cord's central gray matter most often. MRI [1] patients with LETM who test positive for anti-AQP4 antibodies have been shown to have different demographic and clinical characteristics from those who do not [58-60].

We acknowledge that our study has some limitations, including the significant heterogeneity in some analyses; however, this heterogeneity could be explained by the varied data in terms of disease duration, EDSS, country of the study, race of the population, and the used method of MRI. We could not perform a subgroup analysis due to the lack of data. Another limitation is the lack of available data in the included studies regarding the length of time required to reach specific disability levels using the EDSS. Our review has potential limitations related to confounding effects, as the studies included in our analysis have varied populations and study designs. Additionally, the power of our analysis could be affected by the relative sizes and characteristics of the studies included, which may influence the reliability of our findings and should be taken into account when interpreting the results.

## Conclusions

In conclusion, the current evidence suggests that patients with NMOSD are more likely to be females with older age and higher EDSS compared to MS patients. Optic neuritis, myelitis, headache, sensory affection symptoms, and visual involvement symptoms are the most commonly reported symptoms in patients with NMOSD. Patients with NMOSD were associated with a lower risk of developing brain lesions compared to MS patients; however, spinal cord lesions, optic nerve lesions, deep gray matter lesions, deep white matter lesions, hypothalamus lesions, and medulla oblongata lesions were more common in NMOSD patients than MS patients. Dawson's finger-type lesions and S or U-shaped lesions are less frequently found in NMOSD patients. The length of the spinal lesions was significantly higher in the NMOSD groups than in the MS group. The NMOSD group was associated with a lower risk of peripheral lesions and a higher risk of cervicothoracic lesions. Atrophic lesions were found to be significantly higher in the NMOSD group than in the MS group.

## Appendices

**Table 5:** Quality assessment using JBI critical appraisal checklist for cross-sectional studies. JBI: Joanna Briggs Institute.

Study ID		Were the criteria for inclusion in the sample clearly defined?	Were the eight study subjects and the setting described in detail?	Was the exposure measured in a valid and reliable way?	Were objective, standard criteria used for measurement of the condition?	Were confounding factors identified?	Were strategies to deal with confounding factors stated?	Were the outcomes measured in a valid and reliable way?	Was appropriate statistical analysis used?	Overall
Banks, 2020		1	1	1	1	0	0	1	1	6
Kim, 2022		1	1	1	1	1	1	1	1	8
Masuda, 2022		1	1	1	1	1	1	1	1	8
Kumar, 2021		1	1	1	1	0	0	1	1	6
Jang, 2020		1	1	1	1	0	0	1	1	6
Kim, 2020		1	1	1	1	1	1	1	1	8
Lee, 2019		1	1	1	1	1	1	1	1	8
Contentti, 2020		1	1	1	1	1	1	1	1	8
Duan, 2021		1	1	1	1	0	0	1	1	6
Hyun, 2019		1	1	1	1	0	0	1	0	5
Lee, 2018		1	0	1	1	0	0	1	1	5
Wei, 2018		1	1	1	1	0	0	0	1	5
Buch, 2017		0	0	1	1	0	0	1	1	4
Fan, 2017		1	1	1	1	0	0	1	1	6
Hyun, 2017		1	1	1	1	1	1	1	1	8
Viswanathan, 2013		1	1	1	1	0	0	1	0	5
Tatekawa, 2018		1	1	1	1	0	0	1	1	6
Long, 2014		1	1	1	1	0	0	1	1	6
Zhang, 2014		1	1	1	1	0	0	1	1	6
Liao et al., 2014		1	1	1	1	0	0	1	1	6
Lu, 2011		1	1	1	1	0	0	1	1	6

**Table 6:** Quality assessment using JBI critical appraisal checklist for cohort studies. JBI: Joanna Briggs Institute.

Domain	Matthews, 2015	Liu, 2018
Were the two groups similar and recruited from the same population?	1	1
Were the exposures measured similarly to assign people to both exposed and unexposed groups?	1	1
Was the exposure measured in a valid and reliable way?	1	1
Were confounding factors identified?	1	0
Were strategies to deal with confounding factors stated?	1	0
Were the groups/participants free of the outcome at the start of the study (or at the moment of exposure)?	1	0
Were the outcomes measured in a valid and reliable way?	1	1
Was the follow-up time reported and sufficient to be long enough for outcomes to occur?	1	1
Was the follow-up complete, and if not, were the reasons for loss to follow-up described and explored?	1	1
Were strategies to address incomplete follow-up utilized?	0	0
Was appropriate statistical analysis used?	1	1
Overall	10	7
